# Serum ferritin as a potential biomarker for distinguishing advanced-stage colorectal cancer: a retrospective cohort study

**DOI:** 10.7717/peerj.21449

**Published:** 2026-06-17

**Authors:** Chuanpei Cao, Fan Tang, Zhihua Zhu, Feng Wang, Jinfeng Li

**Affiliations:** 1 Jiujiang College Affiliated Hospital, Jiujiang, Jiangxi, China; 2 Jiangxi Dermatology Hospital, Nanchang, Jiangxi, China; 3 Department of Hematology, Jiujiang City Key Laboratory of Cell Therapy, Jiujiang NO.1 People’s Hospital, Jiujiang, Jiangxi, China

**Keywords:** Colorectal cancer, Serum ferritin, Biomarkers, Cancer staging, Staging accuracy

## Abstract

**Background:**

Colorectal cancer (CRC) stage plays a major role in treatment decisions and prognosis. This study investigates whether Serum Ferritin can improve CRC staging and compares its performance to standard markers like carbohydrate antigen 19-9 (CA 19-9) and carcinoembryonic antigen (CEA).

**Methods:**

We analyzed data from 419 CRC patients at Jiujiang College Affiliated Hospital, dividing them into early-stage (I–II, 245 patients) and late-stage (III–IV, 174 patients) groups. Logistic regression and receiver operating characteristic (ROC) curves were used to assess the usefulness of serum ferritin compared with traditional markers.

**Results:**

Serum Ferritin was significantly higher in late-stage CRC (*P* < 0.001). ROC analysis showed Serum Ferritin had a numerically higher area under the curve (AUC) (0.795) compared to CA 19-9 (0.745) and CEA (0.744) at distinguishing between early and advanced stages. The combined model yielded an AUC of 0.890.

**Conclusion:**

Serum Ferritin could be a useful addition to CRC staging, showing a numerically higher AUC than current markers. Adding it to clinical practice may help refine stage assessment and guide treatment decisions. This study supports its potential as a supplementary tool for stage assessment in CRC management.

## Introduction

Colorectal cancer (CRC) remains one of the leading causes of cancer-related deaths worldwide, with increasing incidence rates, particularly in developed countries (Rawla, Sunkara & Barsouk, 2019; Xi & Xu, 2021). Early detection and accurate staging of CRC are pivotal for determining the appropriate treatment strategies and improving patient outcomes (Chakrabarti et al., 2020; Mo et al., 2023). Despite advancements in diagnostic techniques, there is a continuous search for more effective and reliable biomarkers that can aid in the accurate stage assessment and risk stratification of CRC (Beniwal et al., 2023).

Serum biomarkers have been extensively studied for their potential role in cancer management and prognosis. Carcinoembryonic antigen (CEA) and carbohydrate antigen 19-9 (CA 19-9) are currently the most widely used biomarkers for CRC, although their sensitivity and specificity are not sufficient for effective screening and staging (Gao et al., 2018; Lakemeyer et al., 2021). Therefore, the identification of novel biomarkers with greater accuracy in stage differentiation is crucial. Serum Ferritin, a key intracellular iron storage protein, has recently gained attention as a potential biomarker in various malignancies (Moreira, Mesquita & Gomes, 2020; Nie et al., 2022; Tang et al., 2021). Elevated Serum Ferritin levels have been associated with poor prognosis in different cancers, including liver, breast, and lung cancers (Lee, Song & Eo, 2016; Ren et al., 2022; Song et al., 2018). However, its role in CRC has not been thoroughly investigated, particularly in the context of cancer staging (Kim et al., 2022; Zhang et al., 2024).

Inflammation and iron metabolism play significant roles in cancer development and progression (Brown et al., 2020; Guo et al., 2021). Recently, systemic immune-nutritional indices, such as the neutrophil-to-lymphocyte ratio (NLR) and the systemic immune-inflammation index (SII), have demonstrated prognostic value in CRC *via* accessible blood tests (Keskinkilic et al., 2024; Xu et al., 2025). In this context, Serum Ferritin, being an acute phase reactant, may have a clinical implication for stage assessment in CRC, as elevated levels could reflect not only altered iron metabolism but also the inflammatory state associated with cancer progression (Vila Zarate et al., 2022). Understanding the relationship between Serum Ferritin levels and CRC stages could thus provide valuable insights into the disease’s pathophysiology and aid in developing more effective clinical assessment tools.

This study aims to evaluate the utility of Serum Ferritin in differentiating CRC stages and to compare its efficacy with established biomarkers, CEA and CA 19-9. By conducting a retrospective cohort analysis, we seek to provide novel insights into the utility of Serum Ferritin as a biomarker in CRC, potentially contributing to improved staging protocols and patient management strategies.

## Materials and Methods

### Study population and design

This retrospective cohort study included 419 patients diagnosed with CRC at Jiujiang College Affiliated Hospital between January 2019 and December 2022. Patients were stratified into two groups based on the cancer staging: early-stage (Stages I and II, 245 patients) and late-stage (Stages III and IV, 174 patients). Inclusion Criteria: patients aged 18 years or older with a histopathologically confirmed diagnosis of CRC were included. Only patients with complete medical records, including detailed demographic, clinical, and follow-up data, were considered for the study. Exclusion Criteria: patients were excluded if they had a history of other malignancies, had received neoadjuvant therapy before surgical resection (to ensure accurate pathological staging), had incomplete biomarker data, or were participating in other clinical trials that could influence biomarker levels. The reporting of this study conforms to the STROBE statement guidelines for reporting observational studies.

### Data collection and clinical parameters

Demographic and clinical data, including age, gender, family history of CRC, smoking status, alcohol consumption, dietary habits, symptoms at presentation, and treatment history, were extracted from electronic medical records. Importantly, “treatment history” reflects the definitive therapies administered subsequent to the baseline blood draw and initial clinical staging. The data were anonymized and de-identified prior to analysis.

### Biomarker measurement

Fasting venous blood samples were collected at the time of initial clinical diagnosis, prior to any surgical intervention, chemotherapy, or radiotherapy, and analyzed for biomarkers: Serum Ferritin, CA 19-9, and CEA. Serum Ferritin, CA 19-9, and CEA levels were determined using an electrochemiluminescence immunoassay (ECLIA) on an automatic analyzer (Cobas e601, Roche Diagnostics, Mannheim, Germany).

### Statistical analysis

Univariate and multivariate logistic regression models were employed to evaluate the association of demographic and clinical factors with colorectal cancer stages. To evaluate the combined predictive performance, a multivariable logistic regression model was constructed incorporating Serum Ferritin, CA 19-9, and CEA. The predicted probabilities derived from this logistic regression equation were then utilized as the test variable to generate the combined receiver operating characteristic (ROC) curve and assess discriminative accuracy. The area under the curve (AUC), sensitivity, specificity, and optimal cut-off values were computed. Statistical analyses were performed using SPSS software version 25.0 (IBM Corp, Armonk, NY, USA). A *P* value of less than 0.05 was considered statistically significant. The optimal cut-off value for Serum Ferritin was determined using the Youden Index derived from ROC curve analysis, rather than using standard clinical reference ranges, to maximize diagnostic accuracy for stage differentiation.

### Ethical approval

The study protocol was reviewed and approved by the Ethics Committee of Jiujiang College Affiliated Hospital (NO. jjumer-a-2023-0303), and it complied with the principles of the Declaration of Helsinki. Given the retrospective nature of the study and the use of de-identified patient data, the requirement for informed consent was waived.

**Table 1:** Baseline characteristics and biomarker levels of colorectal cancer patients.

**Characteristics**	**Overall**
Age, median (IQR)	58 (45, 74)
Gender, n (%)	
Female	204 (48.7%)
Male	215 (51.3%)
Family_History, n (%)	
Yes	213 (50.8%)
No	206 (49.2%)
Smoking, n (%)	
Yes	204 (48.7%)
No	215 (51.3%)
Alcohol_Consumption, n (%)	
No	222 (53%)
Yes	197 (47%)
Diet, n (%)	
Balanced	143 (34.1%)
High-fat	131 (31.3%)
Vegetarian	145 (34.6%)
Symptoms, n (%)	
Yes	204 (48.7%)
No	215 (51.3%)
Treatment_History, n (%)	
Surgery	112 (26.7%)
Radiation	105 (25.1%)
Chemotherapy	99 (23.6%)
None	103 (24.6%)
Serum_Ferritin, mean ± sd	161.71 ± 33.837
CA_19_9, mean ± sd	21.763 ± 5.9566
CEA, mean ± sd	3.4269 ± 1.148

**Notes.**

IQR Interquartile Range n (%) Number (percentage) of patients Family_History Indicates presence or absence of colorectal cancer in family CA 19-9 Carbohydrate Antigen 19-9 CEA Carcinoembryonic Antigen sd Standard deviation

## Results

### Baseline characteristics and biomarker levels of colorectal cancer patients

In Table 1, the median age of colorectal cancer patients is presented as 58 years, with an interquartile range (IQR) of 45 to 74 years, indicating a wide age distribution among the cohort. The gender distribution is fairly balanced, with 51.3% males and 48.7% females. Approximately half of the patients report a family history of colorectal cancer (50.8%), reflecting a significant hereditary component in the patient population. Smoking and alcohol consumption habits are evenly split among the patients, with both present in slightly less than half of the cases (48.7% and 47%, respectively). Dietary habits vary, with balanced, high-fat, and vegetarian diets represented almost equally among patients. The presence of symptoms (defined as patient-reported complaints such as abdominal pain, hematochezia, or changes in bowel habits) is reported in 48.7% of the patients, while the remaining 51.3% do not exhibit symptoms. Treatment history shows a diverse approach, with surgery (26.7%), radiation (25.1%), and chemotherapy (23.6%) being the primary modalities, alongside a notable proportion of patients (24.6%) who have not undergone any of these treatments. Biomarker analysis reveals an average serum ferritin level of 161.71 with a standard deviation (sd) of 33.837, a CA 19-9 level of 21.763 (±5.9566 sd), and a CEA level of 3.4269 (±1.148 sd), providing insights into the biological markers prevalent in the study group. To determine if the biomarkers provided overlapping information, we performed a Pearson correlation analysis. The results revealed no significant linear relationship between Serum Ferritin and CA 19-9 (*r* = −0.054, *P* = 0.276) or CEA (*r* = −0.002, *P* = 0.976). Furthermore, CA 19-9 and CEA were not significantly correlated with each other (*r* = −0.061, *P* = 0.216). These findings indicate that Serum Ferritin levels are independent of traditional tumor markers in this cohort.

### Comparative analysis of demographic, clinical, and biomarker variables between early and late-stage colorectal cancer patients

**Figure 1:**
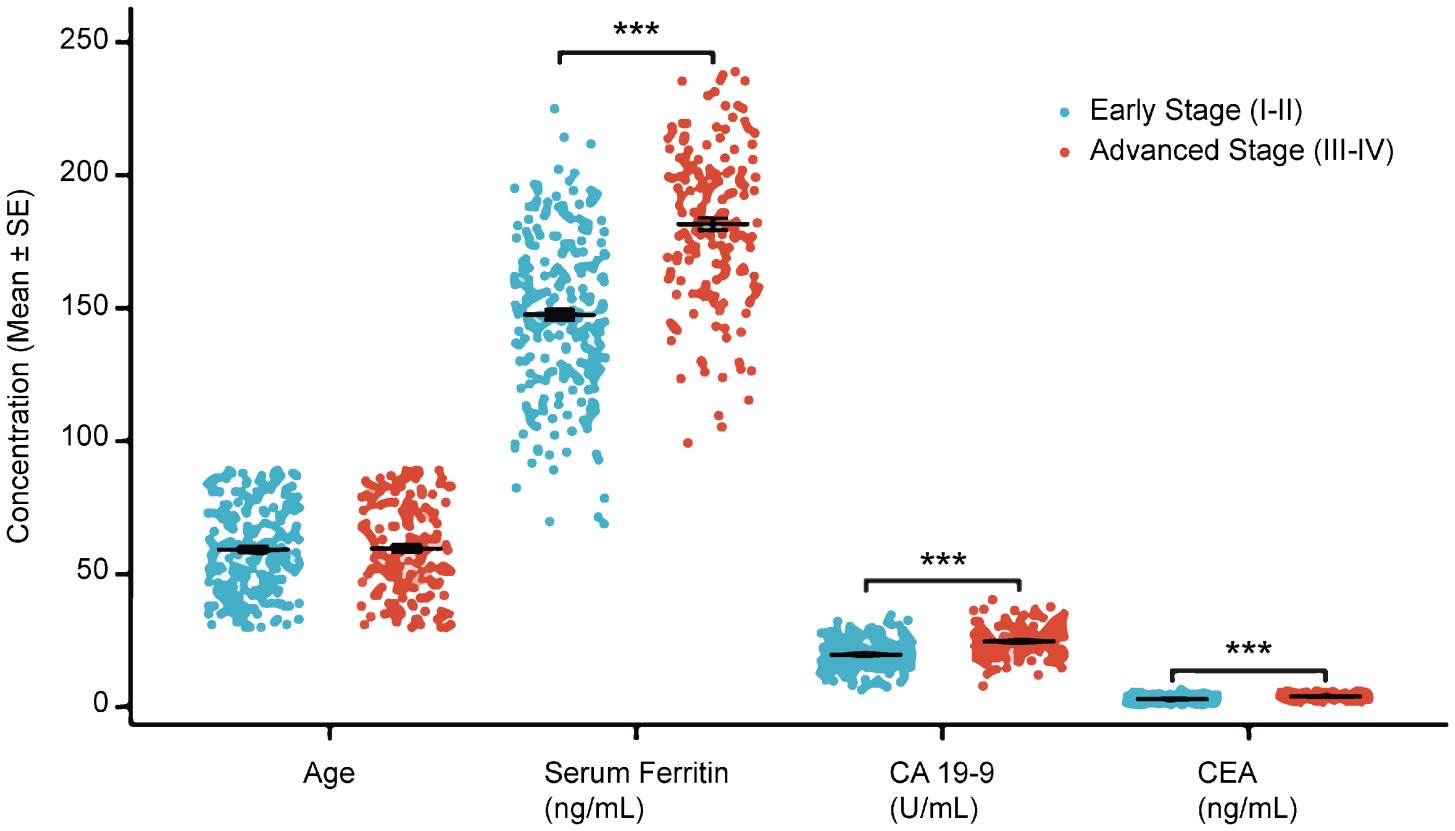
Comparison of biomarker levels between early and late stages of colorectal cancer. This figure illustrates the comparative levels of three biomarkers: Serum Ferritin, CA 19-9, and CEA, in early and late stages of colorectal cancer. The *y*-axis represents the concentration levels of these biomarkers, while the *x*-axis is divided into two major categories: ‘Early’ and ‘Late’ stages of colorectal cancer. Each biomarker is represented by a distinct bar within these categories, indicating the mean concentration levels in each stage. Notably, the graph highlights significant differences in the levels of Serum Ferritin and CEA between early and late stages, as denoted by the asterisks (***), suggesting their potential relevance in the progression and staging of colorectal cancer.

In Table 2, a comprehensive comparative analysis of demographic, clinical, and biomarker variables between early and late-stage colorectal cancer patients is presented. The study involves 245 early-stage and 174 late-stage patients, with median ages closely aligned at 58 and 57.5 years, respectively. Gender distribution across both stages shows a slight predominance of males over females, yet without statistical significance (*P* = 0.395). Family history, smoking habits, alcohol consumption, and diet types (balanced, high-fat, vegetarian) exhibit similar patterns in both early and late-stage groups, with no significant differences noted in their distributions (*P* values ranging from 0.149 to 0.556). Regarding treatment history, surgery and chemotherapy are among the treatments considered, with slightly higher percentages in the early-stage group, although the differences are not statistically significant (*P* = 0.591). However, significant differences are observed in biomarker levels, with Serum Ferritin, CA 19-9, and CEA showing higher mean values in late-stage patients compared to early-stage (*P* < 0.001 for all). Specifically, Serum Ferritin levels average at 147.57 in early-stage and 181.62 in late-stage patients, CA 19-9 levels at 19.676 and 24.701, and CEA levels at 3.022 and 3.997, respectively, indicating a potential correlation of these biomarkers with advanced disease stages (Fig. 1). This analysis provides critical insights into the variations in clinical and biological characteristics between early and late-stage colorectal cancer patients, highlighting the heightened biomarker levels as the disease progresses.

**Table 2:** Comparative analysis of demographic, clinical, and biomarker variables between early and late-stage colorectal cancer patients.

**Characteristics**	**Early**	**Late**	*P* value
n	245	174	
Age, median (IQR)	58 (45, 73)	57.5 (46, 75.75)	0.845
Gender, n (%)			0.395
Female	115 (27.4%)	89 (21.2%)	
Male	130 (31%)	85 (20.3%)	
Family_History, n (%)			0.271
Yes	119 (28.4%)	94 (22.4%)	
No	126 (30.1%)	80 (19.1%)	
Smoking, n (%)			0.149
Yes	112 (26.7%)	92 (22%)	
No	133 (31.7%)	82 (19.6%)	
Alcohol_Consumption, n (%)			0.405
No	134 (32%)	88 (21%)	
Yes	111 (26.5%)	86 (20.5%)	
Diet, n (%)			0.556
Balanced	84 (20%)	59 (14.1%)	
High-fat	72 (17.2%)	59 (14.1%)	
Vegetarian	89 (21.2%)	56 (13.4%)	
Symptoms, n (%)			0.149
Yes	112 (26.7%)	92 (22%)	
No	133 (31.7%)	82 (19.6%)	
Treatment_History, n (%)			0.591
Surgery	64 (15.3%)	48 (11.5%)	
Radiation	64 (15.3%)	41 (9.8%)	
Chemotherapy	53 (12.6%)	46 (11%)	
None	64 (15.3%)	39 (9.3%)	
Serum_Ferritin, mean ± sd	147.57 ± 29.771	181.62 ± 28.887	<0.001
CA_19_9, mean ± sd	19.676 ± 5.3808	24.701 ± 5.4809	<0.001
CEA, mean ± sd	3.022 ± 1.0698	3.997 ± 1.0053	<0.001

**Notes.**

IQR Interquartile Range, representing the range between the 25th and 75th percentiles n (%) Number and percentage of patients in each category *P* value Statistical significance, with values less than 0.05 indicating significant differences between early and late-stage groups sd Standard deviation, indicating the variability around the mean value of biomarkers

### Univariate and multivariate logistic regression analysis of factors associated with advanced-stage colorectal cancer

Table 3 delineates the results from both univariate and multivariate logistic regression analyses, examining various factors associated with advanced-stage colorectal cancer among 419 patients. In the univariate analysis, age did not show a significant association with advanced-stage disease, with an odds ratio (OR) of 1.001 and a *P* value of 0.817. Gender, when compared to females (the reference category), showed that males had a lower, yet non-significant, odds of presenting with advanced-stage disease (OR = 0.845, *P* = 0.396). Similar non-significant associations were observed for family history, smoking, alcohol consumption, diet, and symptoms, indicating no strong univariate relationships with the likelihood of advanced-stage disease. In terms of treatment history, none of the categories, including surgery, radiation, chemotherapy, and no treatment, demonstrated statistically significant associations compared to the surgery group, which served as the reference. However, a notable shift was observed in the biomarker analysis. Serum Ferritin, CA 19-9, and CEA each showed a significant association with late-stage presentation. Specifically, Serum Ferritin and CA 19-9 had odds ratios of 1.040 and 1.186 in the univariate analysis, and 1.040 and 1.166 in the multivariate analysis, respectively, all with *P* values less than 0.001. CEA was notably associated with a higher risk, with an odds ratio of 2.405 in the univariate and 2.266 in the multivariate analysis, also with *P* values less than 0.001. These findings suggest that while demographic and clinical characteristics might not strongly predict colorectal cancer risk individually, certain biomarkers, particularly Serum Ferritin, CA 19-9, and CEA, are significantly associated with an increased likelihood of advanced disease. The multivariate analysis, accounting for multiple factors simultaneously, reaffirms the importance of these biomarkers as potential indicators in colorectal cancer. This comprehensive analysis provides a clearer understanding of the factors linked to colorectal cancer, emphasizing the crucial role of specific biomarkers in predicting the risk of advanced stages.

**Table 3:** Univariate and multivariate logistic regression analysis of factors associated with colorectal cancer.

**Characteristics**	**Total (N)**	**Univariate analysis**		**Multivariate analysis**	
		**Odds ratio (95% CI)**	*P* value	**Odds ratio (95% CI)**	*P* value
Age	419	1.001 (0.990–1.013)	0.817		
Gender	419				
Female	204	Reference			
Male	215	0.845 (0.573–1.247)	0.396		
Family_History	419				
Yes	213	Reference			
No	206	0.804 (0.545–1.186)	0.272		
Smoking	419				
Yes	204	Reference			
No	215	0.751 (0.508–1.108)	0.149		
Alcohol_Consumption	419				
No	222	Reference			
Yes	197	1.180 (0.799–1.741)	0.405		
Diet	419				
Balanced	143	Reference			
High-fat	131	1.167 (0.723–1.883)	0.528		
Vegetarian	145	0.896 (0.559–1.436)	0.648		
Symptoms	419				
Yes	204	Reference			
No	215	0.751 (0.508–1.108)	0.149		
Treatment_History	419				
Surgery	112	Reference			
Radiation	105	0.854 (0.497–1.469)	0.569		
Chemotherapy	99	1.157 (0.672–1.994)	0.599		
None	103	0.813 (0.470–1.403)	0.456		
Serum_Ferritin	419	1.040 (1.031–1.049)	**<0.001**	1.040 (1.030–1.051)	**<0.001**
CA_19_9	419	1.186 (1.136–1.238)	**<0.001**	1.166 (1.109–1.225)	**<0.001**
CEA	419	2.405 (1.934–2.990)	**<0.001**	2.266 (1.742–2.948)	**<0.001**

**Notes.**

Total (N) Total number of patients included in the analysis Odds Ratio (95% CI) Odds ratio with 95% confidence interval, used to express the strength of association between characteristics and colorectal cancer *P* value Indicates the statistical significance of the association, with values less than 0.05 considered significant. Reference The baseline category used for comparison in logistic regression analysis Univariate analysis Analysis considering each factor independently Multivariate analysis Analysis considering all factors simultaneously to adjust for potential confounding variables

Bold styling indicates that *P* is less than 0.05.

### Evaluation of individual biomarkers and combined staging prediction model for colorectal cancer

**Figure 2:**
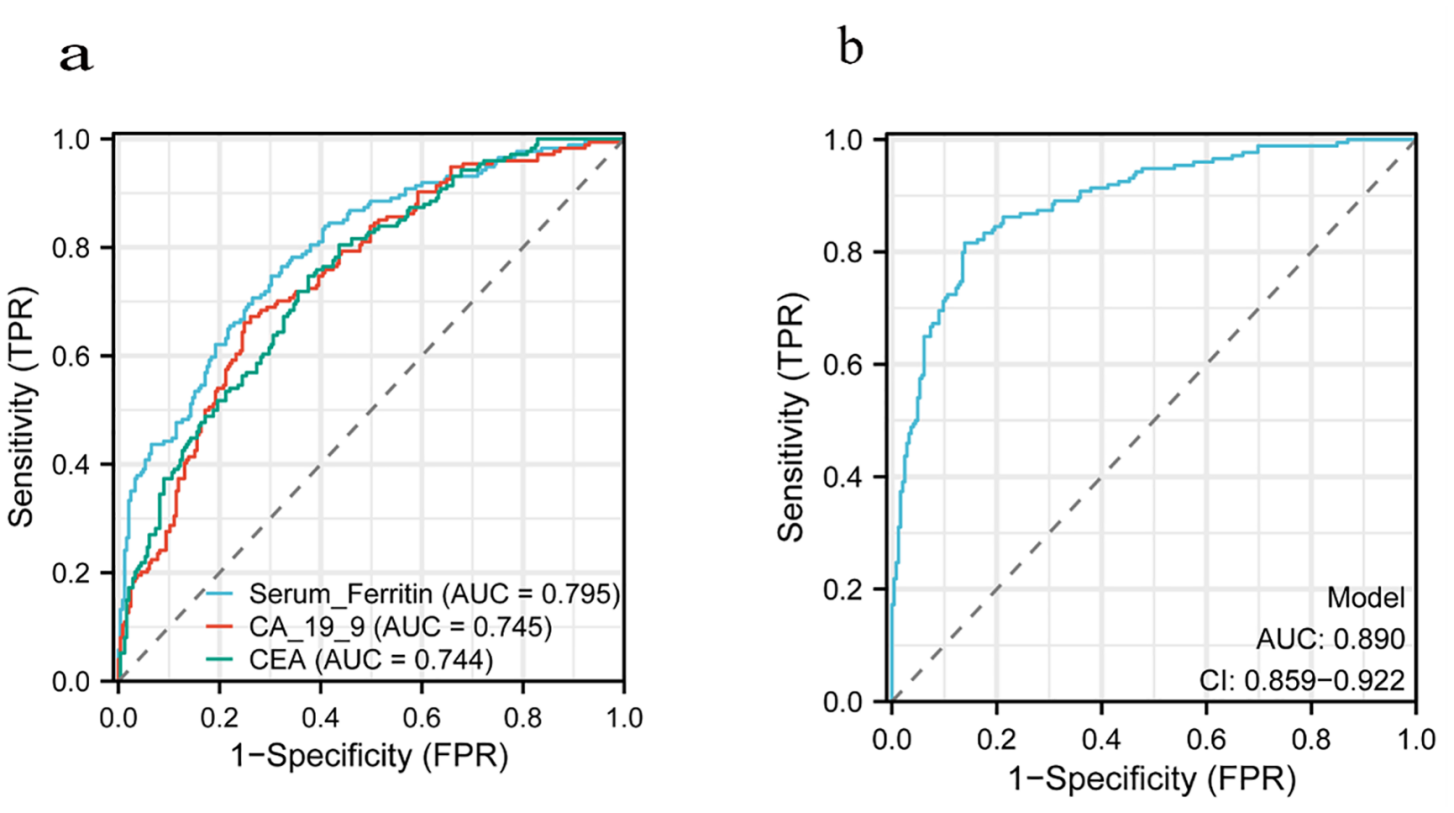
Diagnostic Receiver Operating Characteristic (ROC) curves. Receiver Operating Characteristic (ROC) Curves for Stage Differentiation; (A) Receiver Operating Characteristic (ROC) Curves for Stage Differentiation for Individual Biomarkers in Colorectal Cancer. This figure presents the ROC curves evaluating the performance in distinguishing early *vs.* late stages of three individual biomarkers: Serum Ferritin, CA 19-9, and CEA in colorectal cancer. The *y*-axis represents the sensitivity (True Positive Rate), and the *x*-axis represents 1-Specificity (False Positive Rate). These values indicate the accuracy of each biomarker, with Serum Ferritin showing the highest AUC value, suggesting its superior performance in stage differentiation in CRC compared to CA 19-9 and CEA. (B) Diagnostic ROC Curve for the Combined Biomarker Model in Colorectal Cancer. This figure displays the ROC curve for a combined biomarker model in the stage assessment of colorectal cancer. The *y*-axis represents the sensitivity, and the *x*-axis represents 1-specificity. The ROC curve illustrates the discriminative performance of the combined model, with an AUC of 0.890 and a confidence interval (CI) of 0.859–0.922. The high AUC value indicates the enhanced accuracy in stage prediction of this combined biomarker model, suggesting its potential utility in improving CRC clinical staging.

In Table 4, the diagnostic performance of individual biomarkers, Serum Ferritin, CA 19-9, and CEA, as well as a combined predictive model for CRC staging, is systematically evaluated. The assessment is quantified using the AUC from ROC analysis, with each parameter’s discriminative accuracy between stages encapsulated in these AUC values. Serum Ferritin showed the highest AUC value of 0.795, followed by CA 19-9 (0.745) and CEA (0.744), demonstrating a numerically higher AUC for stage discrimination by Serum Ferritin (Fig. 2A). Serum Ferritin demonstrates a notable AUC of 0.795, within a 95% confidence interval (CI) of 0.751 to 0.838, suggesting a robust capability to identify advanced stages, and a cut-off value is set at 163.32 with specificity and sensitivity rates of approximately 69.8% and 74.7%, respectively. CA 19-9 follows closely with an AUC of 0.745 (95% CI [0.698–0.792]), a cut-off value of 22.859, and specificity and sensitivity of around 75.1% and 66.1%. CEA shows a similar pattern with an AUC of 0.744 (95% CI [0.697–0.790]), a cut-off value of 3.3461, and corresponding specificity and sensitivity of 62.4% and 74.7%. The combined model, however, stands out with an AUC of 0.890, indicating superior predictive accuracy for staging (Fig. 2B). Its cut-off value is set at 0.11996, yielding high specificity and sensitivity rates of 86.1% and 81.6%, respectively. This model, integrating the individual biomarkers, evidently enhances the precision of stage assessment for colorectal cancer, as reflected by its higher AUC and balanced specificity-sensitivity profile. These results highlight the efficacy of both individual biomarkers and the integrated model in stage assessment for colorectal cancer, with the combined model showing a promising potential in clinical applications for clinical stage evaluation.

**Table 4:** Evaluation of individual biomarkers and combined diagnostic model for colorectal cancer.

**Parameters**	**AUC**	**95% CI**	**Cut-off value**	**Specificity (%)**	**Sensitivity (%)**
Serum_Ferritin	0.795	0.751–0.838	163.32	0.69796	0.74713
CA_19_9	0.745	0.698–0.792	22.859	0.75102	0.66092
CEA	0.744	0.697–0.790	3.3461	0.62449	0.74713
Model	0.890	0.859–0.922	0.11996	0.86122	0.81609

**Notes.**

Parameters This column includes individual biomarkers (Serum Ferritin, CA 19-9, CEA) and a combined diagnostic model for colorectal cancer AUC (Area Under the Curve) Represents the diagnostic accuracy of the biomarkers and the model. A higher AUC indicates greater accuracy 95% CI (Confidence Interval) This interval provides a range of values within which the true AUC is likely to fall, offering a measure of reliability Cut-off value The threshold value at which the sensitivity and specificity are optimized for each biomarker and the model Specificity (%) Indicates the percentage of true negatives correctly identified, reflecting the ability to correctly identify patients without colorectal cancer. Sensitivity (%) Represents the percentage of true positives correctly identified, showing the effectiveness in correctly diagnosing patients with colorectal cancer

## Discussion

Our investigation into the staging of CRC reveals a significant elevation in Serum Ferritin (SF) levels in advanced stages, aligning with emerging research in oncological biomarkers, yet presenting a novel perspective in CRC. Unlike previous studies that primarily focused on traditional markers like CEA and Carbohydrate Antigen 19-9 (CA 19-9) (Lee, Teng & Shelat, 2020; Liu et al., 2023; Roh et al., 2017; Xu et al., 2022), our findings underscore SF as a potentially more reliable indicator of CRC progression. This divergence from established diagnostic norms not only challenges the existing paradigm but also enriches our understanding of the biomarker landscape in CRC. For instance, while SF’s role in iron metabolism and inflammatory response is documented in various cancers (Dahman et al., 2022; Shang et al., 2022; Wu et al., 2021; Xue, Deng & Sun, 2019), its specific application in CRC, as our study suggests, could redefine the approach to cancer staging. Mechanistically, iron is essential for cell proliferation, and cancer cells often exhibit an ‘iron addiction’ phenotype with upregulated intracellular ferritin expression to store iron for DNA replication. Elevated serum ferritin in advanced stages may thus reflect both the increased tumor burden (release from necrotic tumor cells) and the systemic inflammatory response.

When contrasting our results with existing literature, the distinct correlation between SF levels and CRC severity emerges as a notable divergence. Previous research on CRC biomarkers has highlighted the variability and limitations of traditional markers like CEA and CA 19-9 across different CRC stages. A study examining the prognostic values of CEA, CA19-9, and CA72-4 in CRC patients indicated variability in their levels and correlation with patient prognoses (Wu, Mo & Wu, 2020). Another investigation discussed the dynamic monitoring of perioperative CEA in CRC, highlighting the controversial benefits of additional measurements of CA19-9 (Li et al., 2021). Further research questioned the independent prognostic significance of preoperative serum CA19-9 in CRC, pointing to the need for more clarity on its interactions with CEA (Li et al., 2022). Moreover, CEA and CA19-9 have been identified as markers more indicative of late stages of carcinogenesis, particularly in cases with developed metastases (Vukobrat-Bijedic et al., 2013). Additionally, a study found a significant correlation between tumor node metastasis (TNM) stages and positive values for both CEA and CA 19-9, yet no significant correlation with other clinical factors, suggesting their limited utility across various CRC stages (Sisik et al., 2013). In contrast, our study demonstrates a more consistent and significant increase in SF levels in advanced CRC, suggesting that SF may offer a more reliable indicator of tumor progression, especially in the later stages of CRC, compared to the traditional markers. This observation is crucial, as it suggests that SF, unlike traditional markers, may more accurately reflect the tumor’s biological behavior and could thus offer a more reliable tool for staging and possibly prognosis.

Additionally, our study was limited by the binary classification of staging (Early *vs.* Late) due to the retrospective nature of data collection, which prevented a more granular analysis of individual TNM stages (I–IV). Furthermore, long-term survival data (*e.g.*, recurrence-free survival) were not available for this cohort. Future prospective studies are needed to evaluate the prognostic value of Serum Ferritin in specific CRC stages.

### Limitations

However, our study has its limitations. First, the retrospective design and the specific patient cohort may introduce selection bias, potentially impacting the generalizability of our findings. Second, due to the retrospective nature of data collection, staging information was aggregated into binary categories (Early *vs.* Late), which prevented a more granular analysis of individual TNM stages (I–IV). Furthermore, long-term follow-up data, specifically recurrence-free survival, were not available for this cohort, limiting our ability to assess the long-term prognostic value of Serum Ferritin. Fourth, as a retrospective study, we were unable to fully account for all potential confounding factors that influence serum ferritin levels, such as pre-existing anemia, oral iron supplementation, or recent blood transfusions, as these data were not systematically available in the dataset. While we excluded patients with other known malignancies, these unmeasured confounders could introduce bias. The lack of parallel investigation into other biomarkers, such as microRNA or DNA alterations, may also limit the comprehensiveness of our understanding of CRC pathophysiology. Future studies, therefore, should aim to include a broader range of biomarkers and detailed staging data in a multicenter, prospective design, enhancing the robustness and applicability of the findings. Fifth, our combined predictive model lacks internal validation techniques, such as bootstrap resampling or cross-validation. Because the model was developed and evaluated within the same cohort, its diagnostic performance (AUC) may be overestimated. Such studies could also explore the longitudinal relationship between SF levels and CRC progression, shedding light on the dynamic changes in biomarkers over time.

Additionally, our findings indicate that SF could be instrumental in distinguishing between early and advanced stages of CRC. Given that accurate differentiation between early and late-stage CRC is pivotal for determining appropriate treatment strategies, the ability of SF to reliably indicate advanced disease could have significant implications for clinical decision-making. This aspect warrants further investigation in future studies, which should focus on the utility of SF in initial risk stratification and its role in guiding personalized treatment plans.

## Conclusion

In conclusion, SF shows potential as an accessible, supplementary biomarker to aid in CRC stage assessment. While it cannot replace established pathological or radiological staging methods, its integration into clinical evaluation protocols, pending robust prospective and multicenter validation may assist in refining risk stratification and guiding patient management.

## Supplemental Information

10.7717/peerj.21449/supp-1 Supplemental Information 1 Anonymized raw data

## Additional Information and Declarations

### Data Availability

The following information was supplied regarding data availability: The raw data is available in the Supplementary File.
